# Assessment of Adherence to Iron Chelation Therapy Among Thalassemia Patients in Palestine

**DOI:** 10.1155/anem/6649477

**Published:** 2025-11-29

**Authors:** Hamzeh Al Zabadi, Zein Ieran, Ibrahim Taha

**Affiliations:** ^1^ Public Health Department, Faculty of Medicine and Health Sciences, An-Najah National University, Nablus, State of Palestine, najah.edu; ^2^ Master Program of Public Health Management, Faculty of Medicine and Health Sciences, An-Najah National University, Nablus, State of Palestine, najah.edu; ^3^ Optometry Department, Arab American University, Ramallah, State of Palestine, aauj.edu

## Abstract

**Background:**

Beta‐thalassemia major (β‐TM) is a severe hereditary blood disorder, common in Palestine due to high consanguinity rates. Lifelong iron chelation therapy (ICT) is vital for managing iron overload from regular transfusions, but adherence remains a major challenge.

**Objective:**

This study investigates factors influencing ICT adherence among β‐TM patients in Palestine, focusing on sociodemographic, clinical, psychological, and healthcare‐related aspects.

**Methods:**

A cross‐sectional study was conducted at the National Thalassemia Center, Nablus, from July 2024 to the end of October 2024, including 120 β‐TM patients aged 3–33. Data were collected through structured interviews using a validated questionnaire covering demographics, disease knowledge, adherence, and satisfaction with healthcare. Adherence was based on missed doses and ferritin levels, analyzed using SPSS V21.0.

**Results:**

62.5% of patients were adherent, with ferritin levels ≤ 2500, while nonadherent patients had levels ≥ 2501 (*p* < 0.001). Although 98.7% of adherent and 100% of nonadherent patients had good disease knowledge, it did not predict adherence. Barriers included psychological distress (21.7%), medication side effects (16.7%), and inconsistent medication supply. Satisfaction with healthcare staff (*p* < 0.001) and socioeconomic status, particularly income (*p* = 0.014), significantly affected adherence.

**Conclusion:**

Adherence is influenced more by psychological, economic, and healthcare service factors than knowledge. A multidisciplinary approach—providing psychological support, stable medication access, and stronger patient–provider relationships—is essential to improve adherence and outcomes for β‐TM patients in Palestine.

## 1. Introduction

β‐Thalassemia results from mutations in the β‐globin gene that impair or abolish the production of β‐globin chains, leading to an imbalance between α‐ and β‐globin synthesis [1]. Globally prevalent, it poses a notable healthcare challenge, particularly in regions such as Palestine, where approximately 801 patients suffer from the condition without effective curative treatment options. The disease exerts a profound financial and social burden on patients, their families, and the healthcare system as a whole. In Palestine, the high rate of consanguineous marriages—estimated at 45% in marriages contracted after 1980—has contributed significantly to the widespread prevalence of the disorder [2].

Beta‐thalassemia major (β‐TM), the most severe form of the disease, is characterized by severe anemia that necessitates lifelong blood transfusions to sustain life. However, repeated transfusions lead to a condition known as chronic iron overload, where excess iron accumulates in vital organs, including the heart and liver [3]. This accumulation is potentially fatal, as it significantly increases the risk of heart failure, stroke, and various endocrine complications [4, 5, 25]. Iron‐induced heart disease remains the leading cause of premature death in individuals with chronic iron overload [6].

To manage iron overload, iron chelation therapy (ICT) is essential. For decades, deferoxamine (DFO) has served as the primary treatment option. Despite its efficacy, the invasive and time‐consuming nature of DFO administration—requiring subcutaneous infusions over 8–12 h multiple times a week—has been a major deterrent to patient adherence [7, 8]. More recently, oral chelators such as deferiprone and deferasirox have emerged as effective alternatives. Deferasirox, in particular, has shown considerable promise due to its once‐daily oral dosing, which improves convenience and adherence [5, 9]. However, like any medication, it comes with challenges, such as potential side effects that require close monitoring [10]. While these advancements in treatment offer hope, adherence to ICT remains a significant obstacle, with personal, social, and treatment‐related factors playing critical roles [11, 12].

The issue of adherence to ICT is multifaceted, involving a range of patient‐related factors, including developmental and psychological challenges. Young patients, who often struggle with balancing dependence and independence, face increased risks of nonadherence. Sociocultural factors, such as family income, parental education, and community support, further compound these challenges [11, 13, 14]. Additionally, the nature of the patient–provider relationship, including the level of trust and support provided by medical staff, has been identified as a critical determinant of adherence rates. These factors highlight the urgent need for comprehensive interventions that address both medical and psychosocial aspects of care [15].

Previous research underscores the importance of psychological and social dimensions in managing chronic conditions. For example, studies on adolescents with Type 1 diabetes have demonstrated how psychological factors such as motivation, social anxiety, and family dynamics significantly influence adherence to treatment regimens and self‐management practices [16]. Similarly, for thalassemia patients, improving adherence requires an integrated approach that considers the impact of social and environmental factors on treatment behaviors. Effective strategies to enhance adherence have been shown to significantly improve the quality of life for patients, reduce complications, and alleviate the broader healthcare burden [17].

Understanding the barriers to ICT adherence is particularly important in the context of Palestine, where limited resources, social stigmas, and gaps in healthcare delivery exacerbate the challenges faced by thalassemia patients. Addressing these barriers not only has the potential to improve individual health outcomes but also to reduce the economic strain on families and the healthcare system. For these reasons, adherence to ICT is a critical area of research that requires urgent attention to improve the lives of those affected by beta‐thalassemia.

This study aims to investigate the factors influencing ICT adherence among beta‐thalassemia patients in Palestine. By identifying patient‐related, medication‐related, sociocultural, and healthcare provider‐related barriers, this research seeks to inform targeted interventions that enhance adherence rates, improve health outcomes, and ultimately reduce the burden of this disease on affected individuals and their communities.

## 2. Methods

### 2.1. Study Design and Settings

A descriptive cross‐sectional design was conducted to evaluate adherence to ICT among thalassemia patients. The research was conducted at the Thalassemia Department of the National Hospital, Nablus, providing access to a centralized population of patients receiving treatment for β‐TM. The study was carried out over a 4‐month period, from July 2024 to end of October 2024.

### 2.2. Study Population and Sampling

A purposive sampling approach was used to include all 120 patients receiving regular treatment at the National Thalassemia Center. The study targeted Palestinian patients aged 3–33 years who had been diagnosed with β‐TM and were receiving ICT with either subcutaneous DFO (Desferal) or oral deferasirox (Exjade).

Although the study primarily focused on patients with β‐TM and intermedia, two participants clinically classified as thalassemia minor were also included. These patients had developed secondary iron overload due to multiple transfusions and were undergoing chelation therapy, meeting the inclusion criterion of “currently receiving iron chelation treatment.”

Patients were excluded if they declined to participate or had unrelated comorbidities, such as malignancy, chronic renal failure, mental illness, or chronic liver disease. However, no patients meeting the inclusion criteria were excluded, and all 120 eligible participants were included in the final analysis.

### 2.3. Data Collection

Data for this study were collected from thalassemia patients through direct interviews or phone calls using a structured questionnaire. Data collection began in July 2024, ensuring comprehensive participation of patients. The questionnaire used in the research consisted of three main sections:

#### 2.3.1. Sociodemographic and Clinical Characteristics

This section gathered demographic and clinical information, including sex, age, place of residence, parental education, patient education, employment status, relationship between parents, family members affected by or deceased from thalassemia, economic status, monthly income, diagnosis, treatment duration, type of ICT, presence of other diseases, and habits such as smoking or other personal management skills.

Social status was determined using a composite of monthly household income, parental education, and employment status. For statistical analysis, families were categorized as low (< 3000 ILS; ≈ < 810 USD), middle (3000–6000 ILS; ≈ 810–1620 USD), or high (> 6000 ILS; > 1620 USD) social status, based on standards of the Palestinian Central Bureau of Statistics.

Comorbidities were recorded as part of the clinical data. “Other diseases” included stable chronic conditions such as hypothyroidism, diabetes mellitus, hepatitis C infection, or cardiac complications related to iron overload.

#### 2.3.2. Assessment of Knowledge Gaps

A 15‐point questionnaire was used to assess knowledge about thalassemia, including the disease itself and complications related to iron overload. Each correct response was scored as 1, uncertain responses as 0.5, and incorrect responses as 0. Knowledge levels were then compared between adherent and nonadherent groups.

#### 2.3.3. Adherence to ICT

Adherence was measured using a Likert scale, where 1 represented “never missed a dose,” 2 indicated “some of the time” (< 25% missed doses), 3 indicated “most of the time” (25%–50% missed doses), and 4 represented “all of the time” (> 50% missed doses). Patients taking > 75% of prescribed doses (scores 1 and 2) were classified as adherent, while those taking < 75% (scores 3 and 4) were nonadherent. Compliance with treatment was assessed across various aspects, including receiving treatment, interactions with medical staff, community integration, and accessing medical services.

#### 2.3.4. Clarification of Dosing and Definition of 100% Adherence

In this study, *“100% adherence”* was defined as no reported missed doses during the preceding month, verified through both patient self‐report and physician documentation.

For DFO, patients were prescribed subcutaneous infusions of 40–50 mg/kg/day, administered over 8–10 h per night for five to six nights per week, in accordance with *Thalassemia International Federation (TIF)* guidelines.

For **deferasirox**, patients received **20–40 mg/kg/day**, taken **once daily on an empty stomach**.

Self‐reported adherence on the Likert scale was cross‐checked with prescribing records to ensure dose and frequency consistency.

The questionnaire was translated into Arabic, adapted for the local context, and validated for use. The full questionnaire is provided as Supporting Information S1.

The questionnaire was validated by experienced specialists with over 20 years of expertise in managing thalassemia patients and reviewed by the dissertation supervisor, along with two other academic public health specialists. Feedback from the Friends of Thalassemia Patients Society provided additional insights into nurse adherence practices and informed question development. Multiple prior studies were reviewed to ensure the tool’s comprehensiveness. To test reliability, a pilot study was conducted before full implementation. Participants reported that the questionnaire was clear and easy to understand, minimizing errors and enhancing data accuracy. Participants were fully informed about the study’s objectives and signed consent forms before participation, ensuring ethical compliance and voluntary participation.

In this study, “hospital treatment” referred to care provided within the National Thalassemia Center, including routine blood transfusions, intravenous or subcutaneous DFO infusions, and medical follow‐up visits. In contrast, “community treatment” referred to care provided at home, primarily the self‐administered oral chelation therapy with deferasirox and associated daily management routines performed outside the hospital. This distinction was important to assess patients’ satisfaction and adherence in both structured clinical and self‐managed environments.

### 2.4. Data Analysis

The questionnaire was reviewed for completeness and consistency before processing. Data were coded, entered, cleaned, and analyzed using the Statistical Package for the Social Sciences (SPSS, Version 21.0). All continuous variables were tested for normality using the Shapiro–Wilk test, with *p* < 0.05 indicating non‐normal distribution. Descriptive statistics were used to summarize the data: Continuous variables were presented as mean ± standard deviation (SD) for normally distributed data and as median (interquartile range, IQR) for non‐normally distributed data, while categorical variables were expressed as frequencies and percentages. Chi‐square (*χ* ^2^) tests were applied to assess associations between categorical variables, and independent‐samples *t*‐tests were used for comparisons of continuous variables where applicable. A *p* value < 0.05 was considered statistically significant.

Ferritin levels were analyzed in two groups:

• **Group 1:** Patients who “never missed a dose” or missed doses “some of the time,” with ferritin levels ≤ 2500 ng/mL.
• **Group 2:** Patients who missed doses “most of the time” or “all of the time,” with ferritin levels ≥ 2501 ng/mL.

### 2.5. Ethical Considerations

The study received postgraduate approval on 19/03/2023 (Appendix A) and ethical approval from the Institutional Review Board (IRB) at An‐Najah National University on 08/05/2023. Official permissions were obtained from Al‐Watani Governmental Hospital to distribute the questionnaire and information sheets in both English and Arabic. Participation was voluntary, with full confidentiality ensured using serial numbers. Participants were informed of their right to withdraw at any time without consequences.

## 3. Results

The study included 120 patients from the Thalassemia Department at Al‐Watani Governmental Hospital, Nablus. In this section of the study, the collected data were tabulated, analyzed, and interpreted individually using statistical programs and tests. Below, this section begins with the characteristics of those studied.

### 3.1. Demographic Characteristics of Participants

A total of 120 thalassemia patients participated in the study and completed the questionnaire. The gender distribution was approximately equal, with 63 males (52.5%) and 57 females (47.5%). The majority of participants (52%) were between 18 and 26 years old.

Regarding treatment, participants were receiving either DFO or deferasirox as ICT. The mean prescribed dose was 45 ± 5 mg/kg/day for DFO (administered five to six nights per week) and 30 ± 8 mg/kg/day for deferasirox, taken once daily on an empty stomach, in accordance with TIF guidelines.

In terms of comorbid conditions, 15.8% of patients were diagnosed with psychological disorders, primarily including anxiety (8.3%), depression (5.0%), and sleep disturbances or insomnia (2.5%). These diagnoses were obtained from patient medical records and verified by the treating physicians at the National Thalassemia Center. This finding reflects the ongoing psychological burden associated with chronic disease management and long‐term ICT.

Adherence to treatment was assessed based on the frequency of missed doses. Participants who reported never missing a dose or missing only occasionally (< 25%) were classified as adherent. Those who reported missing doses frequently (25%–50%) or most of the time (> 50%) were considered nonadherent.

This classification was supported by serum ferritin levels: 75% of adherent patients had ferritin levels ≤ 2500 ng/mL, whereas 45% of nonadherent patients had levels ≥ 2501 ng/mL (see Table 1 for detailed demographic and clinical characteristics).

**Table 1:** Sociodemographic and clinical characteristics of participants.

Variable	Adhered	Not adhered	Total	Chi‐square
	*N* (%)	*N* (%)		*p* value
Ferritin levels				
Less than and/or equal 2500	75 (100)	0 (0.0)	75	< 0.001
Equal and/or above 2501	0 (0.0)	45 (100)	45	
Gender				
Male	42 (66.7)	21 (33.3)	63	0.322
Female	33 (57.9)	24 (42.1)	57	
Age (years) (mean ± SD)	19.253 ± 7.038	21.288 ± 7.393	—	0.135^∗^
Age				
0–8 years	8 (10.7)	3 (6.7)	11	0.064
9–17 years	23 (30.7)	9 (20)	32	
18–26 years	34 (45.3)	18 (40.0)	52	
27–33 years	10 (13.3)	15 (33.3)	25	
Place				
City	18 (54.5)	15 (45.5)	33	0.348
Village	36 (62.1)	22 (37.9)	58	
Camp, refugee	21 (72.4)	8 (27.6)	29	
The questionnaire has been filled out				
Patient himself	50 (61.0)	32 (39.0)	82	0.612
Patient relative	25 (65.8)	13 (34.2)	38	
Relationship between the parents				
No	9 (52.9)	8 (47.1)	17	0.380
Yes	66 (64.1)	37 (35.9)	103	
Father’s education				
Primary	10 (62.5)	6 (37.5)	16	0.788
Preparatory	27 (65.9)	14 (34.1)	41	
Secondary	31 (57.4)	23 (42.6)	54	
Diploma	3 (75.0)	1 (25.0)	4	
Bachelor’s	4 (80.0)	1 (20.0)	5	
Master	0 (0.0)	0 (0.0)	0	
Mother’s education				
Primary	10 (62.5)	6 (37.5)	16	0.486
Preparatory	21 (55.3)	17 (44.7)	38	
Secondary	37 (71.2)	15 (28.8)	52	
Diploma	5 (50.0)	5 (50.0)	10	
Bachelor’s	2 (50.0)	2 (50.0)	4	
Master	0 (0.0)	0 (0.0)	0	
Patients education				
Primary	10 (58.8)	7 (41.2)	17	0.293
Preparatory	13 (61.9)	8 (38.1)	21	
Secondary	30 (63.8)	17 (36.2)	47	
Diploma	4 (36.4)	7 (63.6)	11	
Bachelor’s	18 (75.0)	6 (25.0)	24	
Master	0 (0.0)	0 (0.0)	0	
Marital status				
Married	13 (50.0)	13 (50.0)	26	1.05
Single	62 (66.0)	32 (34.0)	94	
Widower	0 (0.0)	0 (0.0)	0	
Divorce	0 (0.0)	0 (0.0)	0	
Family members				
Less than 3	7 (63.6)	4 (36.4)	11	0.609
(3–5)	37 (59.7)	25 (40.03)	62	
(6–8)	25 (62.5)	15 (37.5)	40	
More than 8	6 (85.7)	1 (14.3)	7	
Job				
Student	33 (64.7)	18 (35.3)	51	0.087
College	8 (72.7)	3 (27.3)	11	
Not studying	5 (31.3)	11 (68.8)	16	
Personal job	21 (67.7)	10 (32.3)	31	
Employee	8 (72.7)	3 (27.3)	11	
Family members affected by thalassemia				
None	28 (75.7)	9 (24.3)	37	0.120
(1–2)	43 (55.8)	34 (44.2)	77	
More than 2	4 (66.7)	2 (33.3)	6	
Family member died of thalassemia				
None	68 (64.8)	37 (35.2)	105	0.176
One or more	7 (46.7)	8 (53.3)	15	
Social and economic status of the family				
Low	6 (100.0)	0 (0.0)	6	0.143
Medium	68 (60.7)	44 (39.3)	112	
High class	1 (50.0)	1 (50.0)	2	
Monthly income/USD				
Less than (604.26)	21 (77.8)	6 (22.2)	27	0.063
More than (604.26)	54 (58.1)	39 (41.9)	93	
Diagnosis				
Thalassemia minor	2 (100.0)	0 (0.0)	2	0.013
Thalassemia medium	38 (76.0)	12 (24.0)	50	
Thalassemia major	35 (51.5)	33 (48.5)	68	
Sickle‐cell anemia—thalassemia	0 (0.0)	0 (0.0)	0	
Iron chelation therapy treatment period				
Less than a year	5 (71.4)	2 (28.6)	7	0.833
(1–3) years	9 (60.0)	6 (40.0)	15	
(3–5) years.	10 (71.4)	4 (28.6)	14	
More than 5 years	51 (60.7)	33 (39.3)	84	
Treatment type of iron chelation therapy				
Oral tablet	73 (61.9)	45 (38.1)	118	0.269
IV	2 (100.0)	0 (0.0)	2	
Any other disease				
No	67 (63.2)	39 (36.8)	106	0.660
Yes	8 (57.1)	6 (42.9)	14	
Do you have bad habits such as smoking and others				
No	61 (61.0)	39 (39.0)	100	0.614

*Note:* Statistically significant at *p* < 0.05.

## 4. Assessment of Disease Knowledge Gaps Among Thalassemia Patients

The analysis of the disease knowledge gaps of thalassemia patients showed that there was a good understanding of the disease among most of the participants. Most of the patients comprising 73.3% claimed that they possessed adequate knowledge of thalassemia. There were also very high percentages of correct responses to the questions on the disease’s genetic nature (93.3%), the significance of consanguineous marriage as a transmitter of the disease (92.5%), and the need for genetic counseling and investigations before marriage (97.5%). Moreover, patients also had good knowledge regarding the need for ICT by 90.0% who correctly answered that it is done for the management of iron overload and the maintenance of health. However, specific gaps in knowledge were highlighted. For example, only 62.5% of patients knew that the signs of thalassemia appear several months post birth and 64.2% knew that excess iron could damage gland function. In addition, 70.8% rightfully said that thalassemia has a negative effect on the quantity of red blood cells produced, while 73.3% correctly associated iron overload with cardiomyopathy. It can be concluded that these patients to a large extent have the relevant knowledge; however, educational outreach programs should be organized to address these issues, particularly the symptom onset and the consequences of iron overload. Such initiatives supported by caregivers and healthcare providers could further increase the patient’s compliance with treatment and health status.

### 4.1. Association of Disease Knowledge and Satisfaction With Treatment in the Community: Sociodemographic and Clinical Characteristics

The analysis of the association between disease knowledge, satisfaction with treatment in the community, and sociodemographic and clinical characteristics among study participants revealed several key findings. The results are presented, which combines the assessment of disease knowledge and satisfaction levels with treatment in the community.

### 4.2. Disease Knowledge and Sociodemographic/Clinical Characteristics

The analysis indicated that factors such as age, gender, parental education level, place of residence, and the number of family members did not significantly influence participants’ knowledge of thalassemia. However, significant correlations were observed with employment status (*p* = 0.041), socioeconomic status (*p* < 0.001), the presence of other diseases (*p* = 0.006), and monthly income (*p* = 0.062). Patients from families with higher economic status demonstrated a better level of disease knowledge, and those without other comorbid conditions showed more advanced understanding. Additionally, students, in particular, were found to have sufficient information about their disease, highlighting the importance of considering social and economic factors in health education efforts aimed at enhancing awareness and improving disease management (Table 2).

**Table 2:** Association of disease knowledge and satisfaction with treatment in the community: sociodemographic and clinical characteristics.

Variable	Association of disease knowledge degree with the study sociodemographic and clinical characteristics among the participants of study subjects			Association of degree of satisfaction when receiving the treatment in community with the study sociodemographic and clinical characteristics among the participants of study subjects		
	Good	Poor	Chi‐square *p* value	Good	Poor	Chi‐square *p* value
Gender						
Male	62 (98.4)	1 (1.6)	0.339	45 (71.4)	18 (28.6)	0.573
Female	57 (100)	0 (0.0)		38 (66.7)	19 (33.3)	
Age						
0–8 years	11 (100)	0 (0.0)	0.428	9 (81.8)	2 (18.2)	0.140
9–17 years	31 (96.9)	1 (3.1)		25 (78.1)	7 (21.9)	
18–26 years	52 (100)	0 (0.0)		36 (69.2)	16 (30.8)	
27–33 years	25 (100)	0 (0.0)		13 (52.0)	12 (48.0)	
Place of residence						
City	32 (97)	1 (3)	0.265	21 (63.6)	12 (36.4)	0.188
Village	58 (100)	0 (0.0)		38 (65.5)	20 (34.5)	
Camp, refugee	29 (100)	0 (0.0)		24 (82.8)	5 (17.2)	
The questionnaire has been filled out						
Patient himself	82 (100)	0 (0.0)	0.140	55 (67.1)	27 (32.9)	0.466
Patient relative	37 (97.4)	1 (2.6)		28 (73.7)	10 (26.3)	
Relationship between the parents						
No	17 (100)	0 (0.0)	0.683	11 (64.7)	6 (35.3)	0.667
Yes	102 (99.0)	1 (1.0)		72 (69.9)	31 (30.1)	
Father’s education						
Primary	16 (100)	0 (0.0)	0.746	11 (68.8)	5 (31.3)	0.715
Preparatory	40 (97.6)	1 (2.4)		31 (75.6)	10 (24.4)	
Secondary	54 (100)	0 (0.0)		34 (63.0)	20 (37.0)	
Diploma	4 (100)	0 (0.0)		3 (75.0)	1 (25.0)	
Bachelor’s	5 (100)	0 (0.0)		4 (80.0)	1 (20.0)	
Mother’s education						
Primary	16 (100)	0 (0.0)	0.858	11 (68.8)	5 (31.3)	0.420
Preparatory	38 (100)	0 (0.0)		24 (63.2)	14 (36.8)	
Secondary	51 (98.1)	1 (1.9)		40 (76.9)	12 (23.1)	
Diploma	10 (100)	0 (0.0)		5 (50.0)	5 (50.0)	
Bachelor’s	4 (100)	0 (0.0)		3 (75.0)	1 (25.0)	
Patients’ education						
Primary	17 (100)	0 (0.0)	0.815	11 (64.7)	6 (35.3)	0.390
Preparatory	21 (100)	0 (0.0)		14 (66.7)	7 (33.3)	
Secondary	46 (97.9)	1 (2.1)		35 (74.5)	12 (25.5)	
Diploma	11 (100)	0 (0.0)		5 (45.5)	6 (54.5)	
Bachelor’s	24 (100)	0 (0.0)		18 (75.0)	6 (25.0)	
Status						
Married	26 (100)	0 (0.0)	0.597	14 (53.8)	12 (46.2)	0.056
Single	93 (98.9)	1 (1.1)		69 (73.4)	25 (26.6)	
Family members						
Less than 3	11 (100)	0 (0.0)	0.569	7 (63.6)	4 (36.4)	0.775
(3–5)	62 (100)	0 (0.0)		43 (69.4)	19 (30.6)	
(6–8)	39 (97.5)	1 (2.5)		27 (67.5)	13 (32.5)	
More than 8	7 (100)	0 (0.0)		6 (85.7)	1 (14.3)	
Job						
Student	51 (100)	0 (0.0)	0.041	36 (70.6)	15 (29.4)	0.188
College	11 (100)	0 (0.0)		8 (72.7)	3 (27.3)	
Not studying	16 (100)	0 (0.0)		7 (43.8)	9 (56.3)	
Personal job	31 (100)	0 (0.0)		23 (74.2)	8 (25.8)	
Employee	10 (90.9)	1 (9.1)		9 (81.8)	2 (18.2)	
Family members affected by thalassemia						
Nobody	37 (100)	0 (0.0)	0.755	30 (81.1)	7 (18.9)	0.095
(1–2)	76 (98.7)	1 (1.3)		48 (62.3)	29 (37.7)	
More than 2	6 (100)	0 (0.0)		5 (83.3)	1 (16.7)	
Family member died of thalassemia						
Nobody	104 (99)	1 (1.0)	0.704	75 (71.4)	30 (28.6)	0.156
One or more	15 (100)	0 (0.0)		8 (53.3)	7 (46.7)	
Social and economic status of the family						
Low	5 (83.3)	1 (16.7)	< 0.001	6 (100)	0 (0.0)	0.211
Medium	112 (100)	0 (0.0)		76 (67.9)	36 (32.1)	
High class	2 (100)	2 (100)		1 (50.0)	1 (50.0)	
Monthly income (USD)						
Less than (604.26)	26 (96.3)	1 (3.7)	0.062	23 (85.2)	4 (14.8)	0.014
More than (604.26)	93 (100)	0 (0.0)		60 (64.5)	33 (35.5)	
Diagnosis						
Thalassemia minor	2 (100)	0 (0.0)	0.494	2 (100)	0 (0.0)	0.107
Thalassemia medium	49 (98)	1 (2.0)		39 (78.0)	11 (22.0)	
Thalassemia major	68 (100)	0 (0.0)		42 (61.8)	26 (38.2)	
Iron chelation therapy treatment period						
Less than a year	7 (100)	0 (0.0)	0.934	6 (85.7)	1 (14.3)	0.729E
(1–3) years	15 (100)	0 (0.0)		11 (73.3)	4 (26.7)	
(3–5) years	14 (100)	0 (0.0)		10 (71.4)	4 (28.6)	
More than 5 years	83 (98.8)	1 (1.2)		56 (66.7)	28 (33.3)	
Treatment type of iron chelation therapy						
Oral tablet	117 (99.2)	1 (0.8)	0.896	81 (68.6)	37 (31.4)	0.341
IV	2 (100)	0 (0.0)		2 (100)	0 (0.0)	
Any other disease						
No	106 (100)	0 (0.0)	0.006	74 (69.8)	32 (30.2)	0.674
Yes	13 (92.9)	1 (7.1)		9 (64.3)	5 (35.7)	
Do you have bad habits such as smoking and others						
No	99 (99.0)	1 (1.0)	0.653	68 (68.0)	32 (32.0)	0.536
Yes	20 (100)	0 (0.0)		15 (75.0)	5 (25.0)	

### 4.3. Satisfaction With Treatment in the Community and Sociodemographic/Clinical Characteristics

The analysis of satisfaction levels with community‐based treatment revealed that variables such as age, gender, parents’ education levels, place of residence, and family size did not significantly impact participants’ satisfaction. However, certain factors showed notable correlations. For instance, the presence of other diseases (*p* = 0.106) and bad habits such as smoking (*p* = 0.206) were identified as significant factors affecting satisfaction. These findings underscore the complexity of factors influencing patient satisfaction in community settings and suggest areas where targeted interventions could enhance patient experiences and outcomes (Table 2).

### 4.4. Association of Satisfaction With Treatment, Medical Services, and Medical Staff in the Hospital: Sociodemographic and Clinical Characteristics

This section combines the analysis of satisfaction levels with treatment, medical services, and medical staff in the hospital, as well as their associations with sociodemographic and clinical characteristics among study participants. The results are presented in Table 3, which integrates the findings from the three analyses.

**Table 3:** Association of satisfaction with treatment, medical services, and medical staff in the hospital: sociodemographic and clinical characteristics.

Variables	Association of degree of satisfaction in receiving the treatment in the hospital with the study sociodemographic and clinical characteristics among the participants of study subjects			Association of degree of satisfaction in receiving the medical service in the hospital with the study sociodemographic and clinical characteristics among the participants of study subjects			Association of degree of satisfaction when with the medical staff in the hospital with the study sociodemographic and clinical characteristics among the participants of study subjects		
	Good	Poor	Chi‐square *p* value	Good	Poor	Chi‐square *p* value	Good	Poor	Chi‐square *p* value
Gender									
Male	57 (90.5)	6 (9.5)	0.855	45 (71.4)	18 (28.6)	0.573	45 (71.4)	18 (28.6)	0.121
Female	51 (89.5)	6 (10.5)		38 (66.7)	19 (33.3)		33 (57.9)	24 (42.1)	
Gender									
0–8 years	11 (100)	0 (0.0)	0.686	9 (81.8)	2 (18.2)	0.140	9 (81.8)	2 (18.2)	0.014
9–17 years	29 (90.6)	3 (9.4)		25 (78.1)	7 (21.9)		25 (78.1)	7 (21.9)	
18–26 years	46 (88.5)	6 (11.5)		36 (69.2)	16 (30.8)		34 (65.4)	18 (34.6)	
27–33 years	22 (88)	3 (12)		13 (52.0)	12 (48.0)		10 (40.0)	15 (60.0)	
Place									
City	30 (90.9)	3 (9.1)	0.735	21 (63.6)	12 (36.4)	0.188	18 (54.5)	15 (45.5)	0.213
Village	51 (87.9)	7 (12.1)		38 (65.5)	20 (34.5)		38 (65.5)	20 (34.5)	
Camp, refugee	27 (93.1)	2 (6.9)		24 (82.8)	5 (17.2)		22 (75.9)	7 (24.1)	
The questionnaire has been filled out									
Patient himself	73 (89.0)	9 (11.0)	0.601	55 (67.1)	27 (32.9)	0.466	51 (62.2)	31 (37.8)	0.344
Patient relative	35 (92.1)	3 (7.9)		28 (73.7)	10 (26.3)		27 (71.1)	11 (28.9)	
Relationship between the parents									
No	16 (94.1)	1 (5.9)	0.541	11 (64.7)	6 (35.3)	0.667	9 (52.9)	8 (47.1)	0.261
Yes	92 (89.3)	11 (10.7)		72 (69.9)	31 (30.1)		69 (67.0)	34 (33.0)	
Father’s education									
Primary	13 (81.3)	3 (18.8)	0.663	11 (68.8)	5 (31.3)	0.715	10 (62.5)	6 (37.5)	0.719
Preparatory	37 (90.2)	4 (9.8)		31 (75.6)	10 (24.4)		29 (70.7)	12 (29.3)	
Secondary	49 (90.7)	5 (9.3)		34 (63.0)	20 (37.0)		32 (59.3)	22 (40.7)	
Diploma	4 (100)	0 (0.0)		3 (75.0)	1 (25.0)		3 (75.0)	1 (25.0)	
Bachelor’s	5 (100)	0 (0.0)		4 (80.0)	1 (20.0)		4 (80.0)	1 (20.0)	
Mother’s education									
Primary	16 (100)	0 (0.0)	0.116	11 (68.8)	5 (31.3)	0.420	10 (62.5)	6 (37.5)	0.180
Preparatory	33 (86.8)	5 (13.2)		24 (63.2)	14 (36.8)		21 (55.3)	17 (44.7)	
Secondary	48 (92.3)	4 (7.7)		40 (76.9)	12 (23.1)		40 (76.9)	12 (23.1)	
Diploma	7 (70)	3 (30.0)		5 (50.0)	5 (50.0)		5 (50.0)	5 (50.0)	
Bachelor’s	4 (100)	0 (0.0)		3 (75.0)	1 (25.0)		2 (50.0)	2 (50.0)	
Patient’s education									
Primary	15 (88.2)	2 (11.8)	0.770	11 (64.7)	6 (35.3)	0.390	11 (64.7)	6 (35.3)	0.281
Preparatory	19 (90.5)	2 (9.5)		14 (66.7)	7 (33.3)		14 (66.7)	7 (33.3)	
Secondary	42 (89.4)	5 (10.6)		35 (74.5)	12 (25.5)		31 (66.0)	16 (34.0)	
Diploma	9 (81.8)	2 (18.2)		5 (45.5)	6 (54.5)		4 (36.4)	7 (63.6)	
Bachelor’s	23 (95.8)	1 (4.2)		18 (75.0)	6 (25.0)		18 (75.0)	6 (25.0)	
Status									
Married	22 (84.6)	4 (15.4)	0.301	14 (53.8)	12 (46.2)	0.056	13 (50.0)	13 (50.0)	0.070
Single	86 (91.5)	8 (8.5)		69 (73.4)	25 (26.6)		65 (69.1)	29 (30.9)	
Family members									
Less than 3	11 (100)	0 (0.0)	0.374	7 (63.6)	4 (36.4)	0.775	7 (63.6)	4 (36.4)	0.198
(3–5)	56 (90.3)	6 (9.7)		43 (69.4)	19 (30.6)		37 (59.7)	25 (40.3)	
(6–8)	34 (85.0)	6 (15)		27 (67.5)	13 (32.5)		27 (67.5)	13 (32.5)	
More than 8	7 (100)	0 (0.0)		6 (85.7)	1 (14.3)		7 (100)	0 (0.0)	
Job									
Student	45 (88.2)	6 (11.8)	0.650	36 (70.6)	15 (29.4)	0.188	36 (70.6)	15 (29.4)	0.011
College	11 (100)	0 (0.0)		8 (72.7)	3 (27.3)		8 (72.7)	3 (27.3)	
Not studying	15 (93.8)	1 (6.3)		7 (43.8)	9 (56.3)		4 (25.0)	12 (75.0)	
Personal job	28 (90.3)	3 (9.7)		23 (74.2)	8 (25.8)		22 (71.0)	9 (29.0)	
Employee	9 (81.8)	2 (18.2)		9 (81.8)	2 (18.2)		8 (72.7)	3 (27.3)	
Family members affected by thalassemia									
None	34 (91.9)	3 (8.1)	0.589	30 (81.1)	7 (18.9)	0.095	28 (75.7)	9 (24.3)	0.123
(1‐2)	68 (88.3)	9 (11.7)		48 (62.3)	29 (37.7)		45 (58.4)	32 (41.6)	
More than 2	6 (100)	0 (0.0)		5 (83.3)	1 (16.7)		5 (83.3)	1 (16.7)	
Family member died of thalassemia									
None	94 (89.5)	11 (10.5)	0.645	75 (71.4)	30 (28.6)	0.156	69 (65.7)	36 (34.3)	0.434
One or more	14 (93.3)	1 (6.7)		8 (53.3)	7 (46.7)		9 (60.0)	6 (40.0)	
Social and economic status of the family									
Low	6 (100)	0 (0.0)	0.121	6 (100)	0 (0.0)	0.211	6 (100)	0 (0.0)	0.169
Medium	101 (90.2)	11 (9.8)		76 (67.9)	36 (32.1)		71 (63.4)	41 (36.6)	
High class	1 (50)	1 (50)		1 (50.0)	1 (50.0)		1 (50.0)	1 (50.0)	
Monthly income (USD§)									
Less than (604.26)	25 (92.6)	2 (7.4)	0.610	23 (85.2)	4 (14.8)	0.014	21 (77.8)	6 (22.2)	0.114
More than (604.26)	83 (89.2)	10 (10.8)		60 (64.5)	33 (35.5)		57 (61.3)	36 (38.7)	
Diagnosis									
Thalassemia minor	2 (100)	0 (0.0)	0.760	2 (100)	0 (0.0)	0.107	2 (100)	0 (0.0)	0.017
Thalassemia medium	44 (88)	6 (12)		39 (78.0)	11 (22.0)		39 (78.0)	11 (22.0)	
Thalassemia major	62 (91.2)	6 (8.8)		42 (61.8)	26 (38.2)		37 (54.4)	31 (45.6)	
Iron chelation therapy treatment period									
Less than a year	7 (100)	0 (0.0)	0.091	6 (85.7)	1 (14.3)	0.729	6 (85.7)	1 (14.3)	0.369
(1–3) years	11 (73.3)	4 (26.7)		11 (73.3)	4 (26.7)		10 (66.7)	5 (33.3)	
(3–5) years.	12 (85.7)	2 (14.3)		10 (71.4)	4 (28.6)		11 (78.6)	3 (21.4)	
More than 5 years	78 (92.9)	6 (7.1)		56 (66.7)	28 (33.3)		51 (60.7)	33 (39.3)	
Treatment type of iron chelation therapy									
Oral tablet	106 (89.8)	12 (10.2)	0.635	81 (68.6)	37 (31.4)	0.341	76 (64.4)	42 (35.6)	0.295
IV	2 (100)	0 (0.0)		2 (100)	0 (0.0)		2 (100)	0 (0.0)	
Any other disease									
No	96 (90.6)	10 (9.4)	0.570	74 (69.8)	32 (30.2)	0.674	69 (65.1)	37 (34.9)	0.952
Yes	12 (85.7)	2 (14.3)		9 (64.3)	5 (35.7)		9 (64.3)	5 (35.7)	
Do you have bad habits such as smoking and others									
No	89 (89)	11 (11)	0.414	68 (68.0)	32 (32.0)	0.536	63 (63.0)	37 (37.0)	0.304
Yes	19 (95)	1 (5.0)		15 (75.0)	5 (25.0)		15 (75.0)	5 (25.0)	

### 4.5. Satisfaction With Treatment in the Hospital

The analysis revealed that factors such as age, gender, parental education level, place of residence, and the number of family members did not significantly influence participants’ satisfaction with hospital treatment. However, significant correlations were observed with socioeconomic status (*p* = 0.121) and the duration of ICT (*p* = 0.091). Patients with longer treatment periods demonstrated better adherence and understanding of the risks associated with nonadherence. Additionally, patients from families with higher economic status showed higher satisfaction levels, emphasizing the importance of considering social and economic factors in health education efforts aimed at improving medication adherence.

### 4.6. Satisfaction With Medical Services in the Hospital

The analysis of satisfaction with medical services in the hospital indicated that variables such as age, gender, parents’ education levels, place of residence, and family size did not significantly impact satisfaction levels. However, notable correlations were observed with marital status (*p* = 0.056), monthly income (*p* = 0.014), and the number of family members affected by thalassemia (*p* = 0.095). Married patients and those with higher monthly income reported higher satisfaction levels. Furthermore, families with multiple thalassemia patients demonstrated a clearer understanding of the necessity of medication adherence, highlighting the role of familial experience in shaping satisfaction with medical services.

### 4.7. Satisfaction With Medical Staff in the Hospital

The analysis of satisfaction with medical staff in the hospital revealed that factors such as age, gender, parents’ education levels, place of residence, and family size did not significantly influence satisfaction levels. However, significant correlations were observed with the patient’s job status (*p* = 0.011), marital status (*p* = 0.070), and the type of thalassemia diagnosis (*p* = 0.017). Students and employed patients reported higher satisfaction levels, while patients with thalassemia major expressed lower satisfaction compared to those with thalassemia minor or medium. These findings suggest that targeted interventions addressing the specific needs of patients with severe thalassemia and those in certain employment or marital statuses could enhance satisfaction with medical staff.

### 4.8. Assessment of Variables for Patients With Adherence and Nonadherence to Iron Chelation in Hospital

Evaluation of variables for patients with adherence and nonadherence to ICT was conducted in the hospital. In this table, patient variables were classified based on adherence and nonadherence to medication and categorized into “good” and “poor.”

A survey was conducted to assess patients’ knowledge about thalassemia and ICT. This section consisted of 15 questions. In the analysis, patients who answered “yes” were assigned 1 point, while those who answered “no” received 0 points. A cutoff point of 6 was used, and patients were classified into two groups based on their scores: Scores ranging from 0 to 6 were considered poor, while scores greater than 6 were considered good. The study found that 98.7% of adherent patients gave good answers, whereas 100% of nonadherent patients gave good answers. Only 1.3% of adherent patients gave poor answers, while none of the nonadherent patients scored poorly.

A survey was also conducted to assess satisfaction with receiving hospital treatment among thalassemia patients. This section consisted of 8 questions. In the analysis, patients who answered “yes” were assigned 1 point, while those who answered “no” received 0 points. A cutoff point of 2 was used, and patients were classified into two groups: Scores ranging from 0 to 2 were considered poor, while scores greater than 2 were considered good. The study found that 98.7% of adherent patients gave good answers, whereas 75.6% of nonadherent patients gave good answers. Only 1.3% of adherent patients gave poor answers, while 24.4% of nonadherent patients gave poor answers.

A survey was also conducted to assess patient satisfaction with ICT in relation to community and family support. This section consisted of 6 questions. In the analysis, patients who answered “yes” were assigned 1 point, while those who answered “no” received 0 points. A cutoff point of 3 was used, and patients were classified into two groups: Scores ranging from 0 to 3 were considered poor, while scores greater than 3 were considered good. The study found that 82.7% of adherent patients gave good answers, whereas 28.9% of nonadherent patients gave good answers. Only 17.3% of adherent patients gave poor answers, while 71.1% of nonadherent patients gave poor answers.

A further survey was conducted to assess patient satisfaction with the medical staff at the hospital. This section consisted of 16 questions. In the analysis, patients who answered “yes” were assigned 1 point, while those who answered “no” received 0 points. A cutoff point of 8 was used, and patients were classified into two groups: Scores ranging from 0 to 8 were considered poor, while scores greater than 8 were considered good. The study found that 98.7% of adherent patients gave good answers, whereas only 8.9% of nonadherent patients gave good answers. Only 1.3% of adherent patients gave poor answers, while 91.1% of nonadherent patients gave poor answers.

In conclusion, the results indicate a strong relationship between patient satisfaction with hospital treatment and their commitment to ICT. Although most patients demonstrated good knowledge of the disease, this factor alone did not significantly affect commitment, as all nonadherent patients also showed good knowledge. Therefore, it is necessary to strengthen programs that improve patients’ satisfaction with hospital medical services and their interactions with medical staff, as well as satisfaction with treatment in the community. Enhancing patient confidence, motivation, and psychosocial support can help increase adherence levels and encourage long‐term commitment to treatment (see Table 4).

**Table 4:** Assessment of variables for patients with adherence and nonadherence to iron chelation in hospital.

Variable	Adhered *N* (%)	Not adhered *N* (%)	Total	Chi‐square *p*value
Disease knowledge				
Good	74 (98.7)	45 (100)	119	0.439
Poor	1 (1.3)	0 (0.0)	1	
Satisfaction receiving the treatment in the hospital				
Good	74 (98.7)	34 (75.6)	108	< 0.001
Poor	1 (1.3)	11 (24.4)	12	
Satisfaction receiving the medical service				
Good	75 (100)	8 (17.8)	83	< 0.001
Poor	0 (0.0)	37 (82.2)	37	
Satisfaction receiving the treatment with community				
Good	62 (82.7)	13 (28.9)	75	< 0.001
Poor	13 (17.3)	32 (71.1)	45	
Satisfaction with the medical staff				
Good	74 (98.7)	4 (8.9)	78	< 0.001
Poor	1 (1.3)	41 (91.1)	42	

### 4.9. Suggestions to Help You Adhere to a Better Way to Take the Medication Continuously

**Figure 1:**
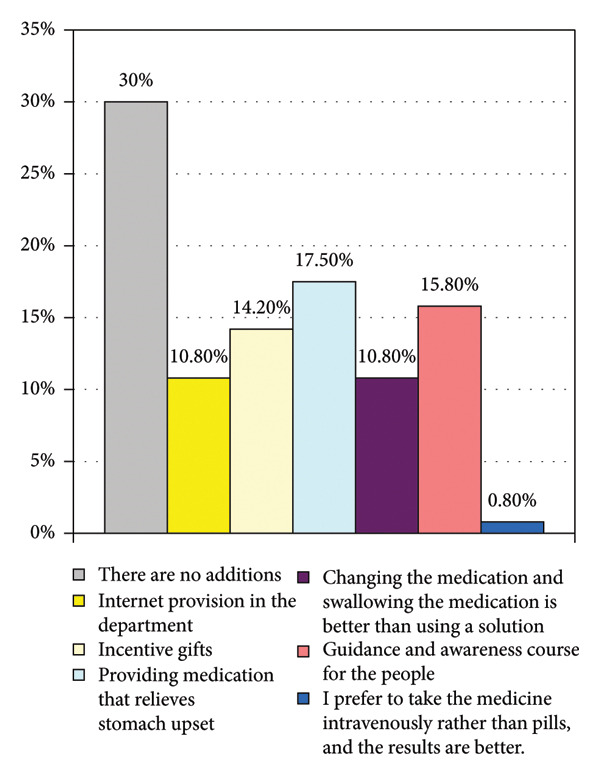
Suggestions to help you adhere to a better way to take the medication continuously.

It describes recommendations made by patients to ensure they follow ICT. Analytics provide solutions. The most prominent of these recommendations is that the majority of patients (17.5%) stated that they need specific medications to reduce the side effects caused by Exjade. Patients also need awareness and counseling sessions. Psychological disorders represented 15.8% of the total, and some individuals said they needed additional support, such as distributing motivational and encouraging gifts to them (see Figure 1).

### 4.10. Barriers to Adherence to ICT Among Thalassemia Patients

**Figure 2:**
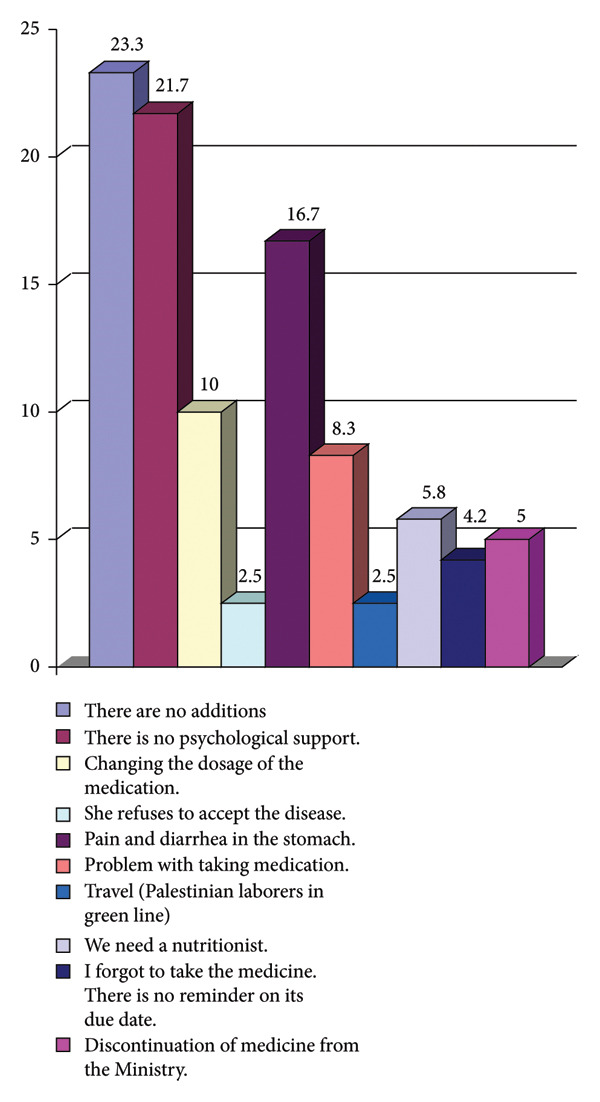
Barriers to adherence to iron chelation therapy among thalassemia patients.

A study showed that many patients do not adhere to taking their medications as prescribed, and an analysis of patients’ answers showed that there are several main reasons for this behavior. The most prominent of which is the urgent need for psychological support, which reached 21.7%. Therefore, support for patients by doctors and healthcare providers, including the provision of psychological support services and psychological counseling, is necessary and has an effective positive impact. Therefore, a group of different adherence techniques must be implemented to improve the patient’s adherence to the treatment prescribed to him. In addition, the pain associated with taking the medication, such as stomach pain and diarrhea, appeared as an influencing factor and accounted for 16.7%. Changing the dosage of the medication also had an impact on patients’ adherence, and its rate was 10% (see Figure 2).

## 5. Discussion

The main study findings are that most participants reported adherence to chelation therapy, while others were nonadherent despite having sufficient knowledge of the disease. The percentage of adherent patients with adequate knowledge was 98.7%, while the percentage of nonadherent patients with sufficient knowledge was 100%. This suggests variety in measurement and definitions for adherence and did not reveal a relationship between their knowledge of thalassemia and their adherence to ICT. While nearly all patients demonstrated good factual knowledge about thalassemia and ICT, this knowledge did not consistently translate into optimal adherence behavior. This indicates that the main gap lies not in cognitive understanding but in the behavioral and psychosocial dimensions of adherence—such as motivation, emotional support, and treatment fatigue. Therefore, the recommendation for increased awareness refers to the need for continuous motivational and behavioral support programs, rather than additional informational education.

It is important to note that, in this study, patients who reported missing doses “some of the time” (< 25%) were classified as adherent based on established adherence definitions used in similar studies. However, from a clinical perspective, even occasional omissions in ICT can result in progressive iron accumulation and adverse outcomes over time. Therefore, this classification should be interpreted with caution, and future research may consider using more stringent adherence criteria or objective monitoring methods to better capture clinically meaningful adherence behavior. The overall adherence rate of approximately 60% observed in this study is consistent with findings from other centers worldwide. Similar adherence levels have been reported among thalassemia patients in studies conducted in Jordan, India, and Malaysia, indicating that suboptimal adherence remains a widespread challenge despite differences in healthcare systems and resource availability. This alignment with international data supports the external validity of our results and underscores the need for global and locally adapted interventions to improve long‐term adherence to ICT.

Thus, healthcare providers should not only educate patients about their disease and its treatment, but also listen to their beliefs in the necessity and concerns about medications, as in a previous study conducted on thalassemia patients in Jordan [18].

Moreover, the type and availability of chelation medication were closely associated with adherence levels. Participants reported several challenges related to medication use, including recurrent changes in dosage (10%) and difficulty in administration (3.8%). Additionally, around 10% of participants spontaneously mentioned experiencing temporary interruptions in receiving their prescribed chelation medication from Ministry of Health (MoH) pharmacies. These interruptions, which emerged from open‐ended responses rather than structured survey items, likely reflect occasional logistical or supply chain disruptions within the public healthcare system. Such issues are often linked to external factors, including funding constraints and movement restrictions related to the broader political situation. Notably, about one‐third of participants expressed concerns about long‐term dependence on ICT and its perceived impact on their daily lives.

Understanding these obstacles is critical for healthcare providers to develop strategies and plans that effectively support patients’ adherence to their treatment regimen. The results of the questionnaire confirmed the pivotal role of the medical team, with 98.7% of patients’ satisfaction with the medical staff and its role in motivating them to adhere to treatment. Ouattara [19] reinforces the idea by highlighting the importance of the doctor–patient relationship in achieving optimal outcomes in terms of satisfaction and adherence to treatment. In addition, Ouattara [19] suggested that providing simple advice can sometimes be enough to enhance treatment compliance [19].

Multidisciplinary approach to patient care means providing care to overcome the disease through different parts of caregivers (psychologists, nurses, doctors, nutritionists) as our study confirmed the necessity of conducting psychological awareness courses and involving psychological and social support, which is considered one of the most important obstacles that prevent patients from adhering to treatment by 21.7%. Some patients expressed the need for additional psychosocial support to help them sustain long‐term adherence. This included requests for motivational activities or gestures of encouragement from healthcare providers and patient associations. These suggestions reflect the emotional and psychological burden of living with a chronic condition that requires continuous, demanding treatment. Importantly, these statements do not indicate a lack of disease understanding, as most patients demonstrated high knowledge levels regarding the risks of nonadherence. Instead, they highlight the need for sustained motivational strategies and community‐based psychosocial support to maintain treatment engagement.

Other studies such as those conducted by Crosby and colleagues suggest that managing the quality of hemoglobinopathies requires a shift in therapeutic practice from a biomedical model to a holistic biopsychosocial model integrated into care [20]. In addition, a previous study confirmed that as most patients with thalassemia major reach adolescence, it is necessary to conduct periodic sessions of psychological and social support, as it is considered a very important part of patient and family management [21]. In addition, the presence of a nutrition specialist to assess dietary patterns and nutritional status is important as some of participants showed that they consume foods that are high in iron without knowing.

Adherence to ICT in general among people with thalassemia is linked to a number of things, including low monthly family income. There was no correlation between knowledge of thalassemia and levels of adherence in our study. Analysis of the degree of disease knowledge in relation to social, demographic, and clinical characteristics was not statistically significant (*p* = 0.062); however, the correlation between satisfaction with receiving medical service received in the hospital and social, demographic, and clinical characteristics was statistically significant (*p* = 0.014). This finding confirms that financial situation improves the patient, consistent with a previous study conducted in Malaysia in the year 2022 which confirmed a close relationship between monthly income and patient commitment, a very important part of patient and family management [22].

Our study showed an association between the degree of satisfaction when dealing with medical staff and patients’ sociodemographic and clinical characteristics. Our study highlighted the necessity of effective communication with healthcare providers and patients, and establishing clear and open communication between them to encourage dialogue about treatment concerns and compliance barriers. This was clear to the patients when analyzing the data, as the percentage of patients’ contributions varied according to the patient’s type of work (student, college, not studying, working private, and employee) with statistical significance (*p* = 0.011). This is what a previous study conducted in London in 2022 showed the importance of providing comprehensive and sufficient information about the importance of treatment, its benefits in reducing iron accumulation, and the potential consequences of noncompliance [23].

In addition, our study showed that some of the obstacles that prevent patients from adhering to treatment are forgetting the medication, and this percentage was 4.2%. Therefore, it is necessary to intervene using technology such as medication reminder apps or wearable devices to remind patients of medication appointments and the need to take them on time. A previous study in London also emphasized the importance of integrating technology into patients’ lives to help them adhere [23].

Our study showed that, when analyzing patient data, the degree of patient satisfaction with the medical staff, the community, service provision, adequate information about the disease, and others. It is focused on certain factors, including the family’s monthly income, economic and social background, the patient’s educational status, nutritional support, and psychological counseling. It was concluded that education is important. It is necessary for patients, as it has been noted that students have a good level of information and have good commitment, as it helps them make the right decisions and help confront problems in a positive way, and they have an ambition to become healthy, and also psychological, emotional, and social health are the aspects that the patient needs most, and this is what was confirmed by a previous study conducted. In India in 2019, education and training contribute to achieving health independence for individuals and lead to improving the quality of life in general, including emotional, social, and psychological health [24].

This study faced several limitations that should be acknowledged, including a shortage of literature in the investigated field, both in Palestine and globally, which limited the ability to compare findings with other studies or draw broader conclusions. Additionally, time constraints posed a challenge, as the study was conducted within a limited timeframe, which may have affected the depth of data collection and analysis. Despite these limitations, the study provides valuable insights into adherence to ICT among thalassemia patients in Palestine. On the other hand, the study has several strengths, as it addresses a critically important health issue—adherence to ICT among thalassemia patients—which is essential for managing this chronic condition and improving patient outcomes. The study comprehensively evaluated adherence by considering a wide range of factors, including patient‐related, drug‐related, social, cultural, and environmental factors, as well as the patient’s relationship with healthcare providers. Furthermore, the study incorporated insights from multiple specialties, such as healthcare providers, psychologists, and nutritionists, to better understand the complex factors influencing adherence. This multidisciplinary approach enhances the study’s relevance and applicability to real‐world clinical settings.

The study concludes that a substantial proportion of patients with β‐thalassemia at the National Governmental Hospital in Nablus, Palestine, demonstrate high adherence rates to ICT. Nevertheless, the findings emphasize the continuing need for enhanced patient education and sustained psychosocial support. These efforts should aim to deepen patients’ understanding of the disease, its complications, the benefits of consistent treatment, and the risks associated with nonadherence. Healthcare providers play a central role in maintaining adherence, and strong, trust‐based relationships between patients and caregivers are crucial. Elements such as effective communication, mutual respect, and the provider’s supportive attitude greatly influence patients’ motivation to adhere to treatment. The study also identified the absence of a comprehensive, multidisciplinary model of care—incorporating physicians, psychologists, nutritionists, and nursing professionals—as a significant gap in patient management. This limitation highlights the necessity of further research to develop, test, and implement interventions that can strengthen adherence and improve quality of life among thalassemia patients. A holistic approach addressing medical, psychological, and social challenges is therefore essential to optimize treatment outcomes and long‐term health.

To facilitate the application of these findings in clinical and policy settings, the main recommendations are summarized in Table 5.

**Table 5:** Summary of recommendations to enhance adherence to iron chelation therapy among thalassemia patients.

Key recommendation	Description/rationale
1. Strengthening Patient Education	Develop structured, interactive educational programs that reinforce understanding of thalassemia, the importance of chelation, and effective self‐management. These should include regular educational sessions, accessible informational materials, and individualized consultations.
2. Improving Access to Medications	Ensure reliable and uninterrupted medication supply through clear national policies, efficient coordination between the Ministry of Health and hospitals, and partnerships with international organizations such as WHO, UNRWA, and the Thalassemia International Federation.
3. Multidisciplinary Care	Establish multidisciplinary teams involving physicians, nurses, psychologists, nutritionists, and social workers to deliver comprehensive, patient‐centered care that addresses medical, nutritional, and psychosocial needs.
4. Community Participation	Strengthen family and community engagement through peer support groups, awareness programs, and patient associations that promote motivation, emotional support, and long‐term adherence.

These four pillars collectively aim to improve adherence behaviors, enhance patient well‐being, and support sustainable, context‐appropriate care models for individuals living with thalassemia in Palestine.

Based on these findings, several recommendations are proposed to overcome the barriers identified and enhance adherence to ICT among patients with thalassemia. Patient education should be strengthened through structured, interactive programs that reinforce understanding of thalassemia, the importance of chelation, and effective self‐management strategies. These programs should include regular educational sessions, accessible informational materials, and individualized consultations tailored to patients’ specific needs.

In the Palestinian context, where access to health services and medications is often disrupted by movement restrictions and political instability, ensuring continuity of care requires adaptive and locally driven strategies. Improving access to medications is therefore equally important, requiring clear national policies and reliable supply systems coordinated between hospitals, pharmacies, and the MoH. Practical approaches—such as centralized procurement, buffer stock systems, and collaboration with international organizations like WHO, UNRWA, and the TIF—can help secure and sustain the availability of essential chelating agents even during crisis periods.

Establishing multidisciplinary care teams that include healthcare providers, psychologists, nutritionists, and other specialists is recommended to deliver holistic care encompassing medical follow‐up, nutritional guidance, psychological counseling, and social support—factors shown to significantly influence adherence and well‐being. Regular monitoring and follow‐up should be implemented to track adherence patterns, address challenges promptly, and maintain ongoing communication between patients and healthcare providers.

Finally, promoting community and family engagement through support groups and peer forums can provide emotional reinforcement and practical coping strategies, encouraging long‐term adherence. Collectively, these recommendations aim to strengthen adherence behaviors, improve patient education, and support sustainable, comprehensive care models that enhance both treatment outcomes and the overall quality of life of individuals with thalassemia, while remaining feasible within the current humanitarian and resource constraints in Palestine.

**Nomenclature**
β‐TM: Beta‐thalassemia major
ICT: Iron chelation therapy
DFO: Deferoxamine
SPSS: Statistical Package for the Social Sciences
IRB: Institutional Review Board
*p*
: *p* value (probability value)
MOH: Ministry of Health
IV: Intravenous
SD: Standard deviation
RBC: Red blood cells.

## Ethics Statement

This study was conducted in accordance with the principles of the Declaration of Helsinki. The study objectives, methodology, and informed consent procedures were reviewed and approved by the Institutional Review Board (IRB) at An‐Najah National University.

## Consent

Informed consent was obtained from all participants involved in the study. This study was carried out as part of a Master’s graduation project, and the full research is archived at An‐Najah National University repository at https://hdl.handle.net/20.500.11888/16212.

## Disclosure

All authors reviewed and approved the final manuscript.

## Conflicts of Interest

The authors declare no conflicts of interest.

## Author Contributions

Hamzeh Al Zabadi and Zein Ieran contributed to all parts of the study, including design, data collection, analysis, and writing. Ibrahim Taha contributed to the study design, data analysis, and writing.

## Funding

No funding was received for this research.

## Supporting Information

Supporting Information S1: Structured questionnaire used for data collection. It includes items related to sociodemographic characteristics, disease knowledge, and adherence to iron chelation therapy. The questionnaire was developed in Arabic and validated for the local context.

## Supporting information


**Supporting Information** Additional supporting information can be found online in the Supporting Information section.

## Data Availability

The data that support the findings of this study are available from the corresponding author upon reasonable request.
