# Supplementary material for: Assessment of Adherence to Iron Chelation Therapy Among Thalassemia Patients in Palestine
Source: Anemia. 2025 Nov 29;2025:6649477. doi: 10.1155/anem/6649477 (PMC12663867; doi:10.1155/anem/6649477)
Supplement: Supplementary file 1 — Supporting Information Additional supporting information can be found online in the Supporting Information section. [file ANEM-2025-6649477-s001.docx]

**
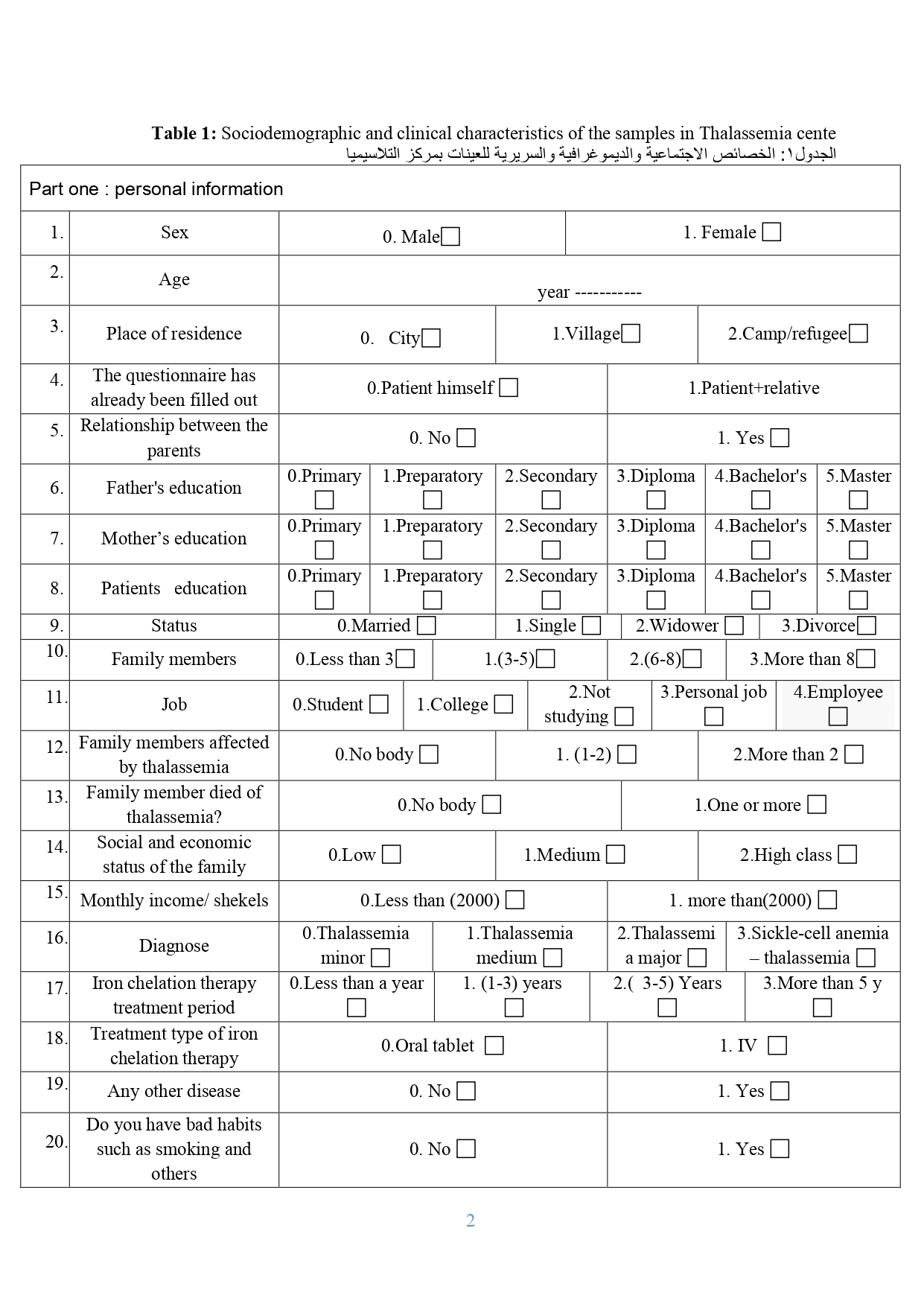
 Supplementary Material S1**

**Questionnaire**


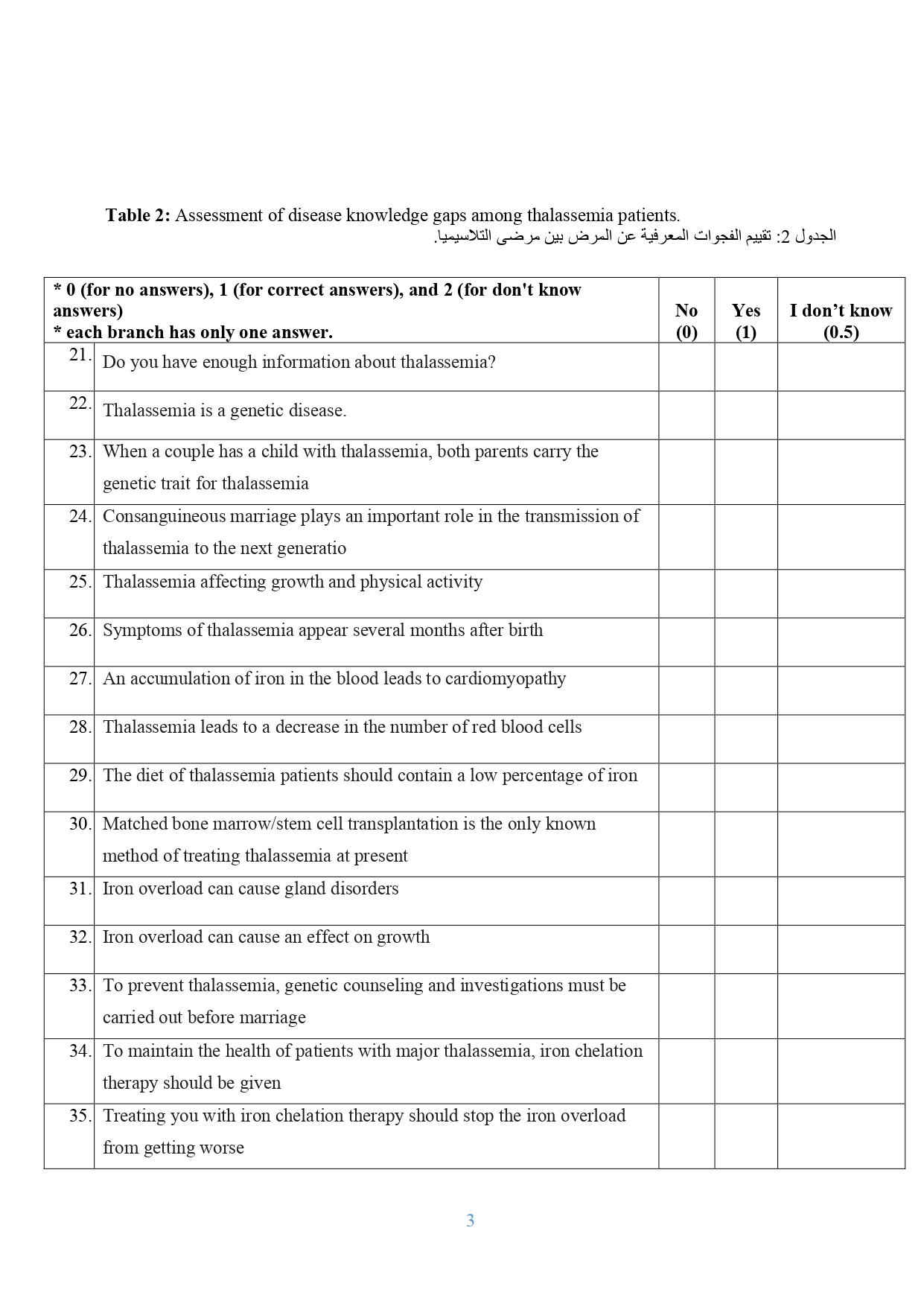


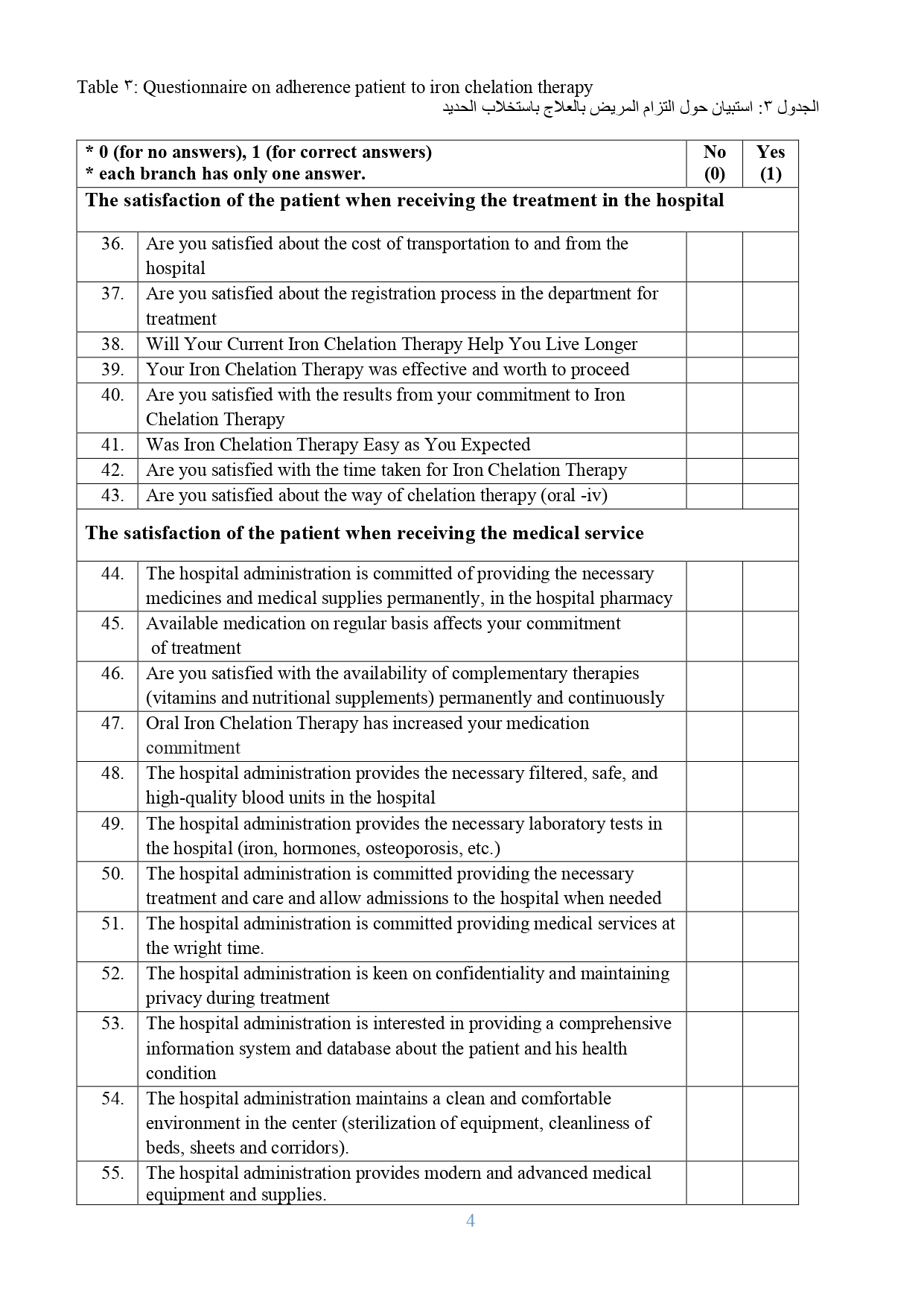


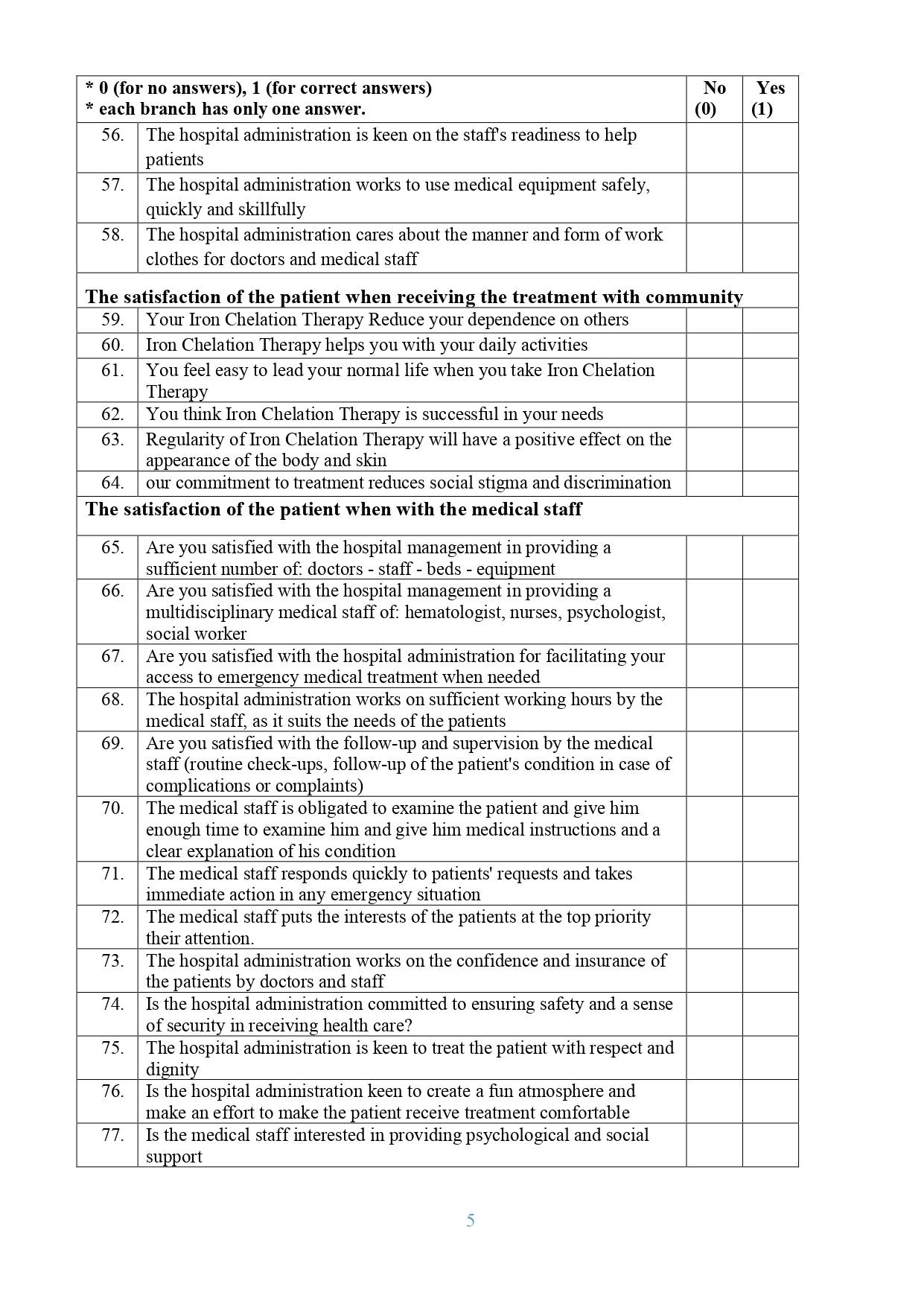


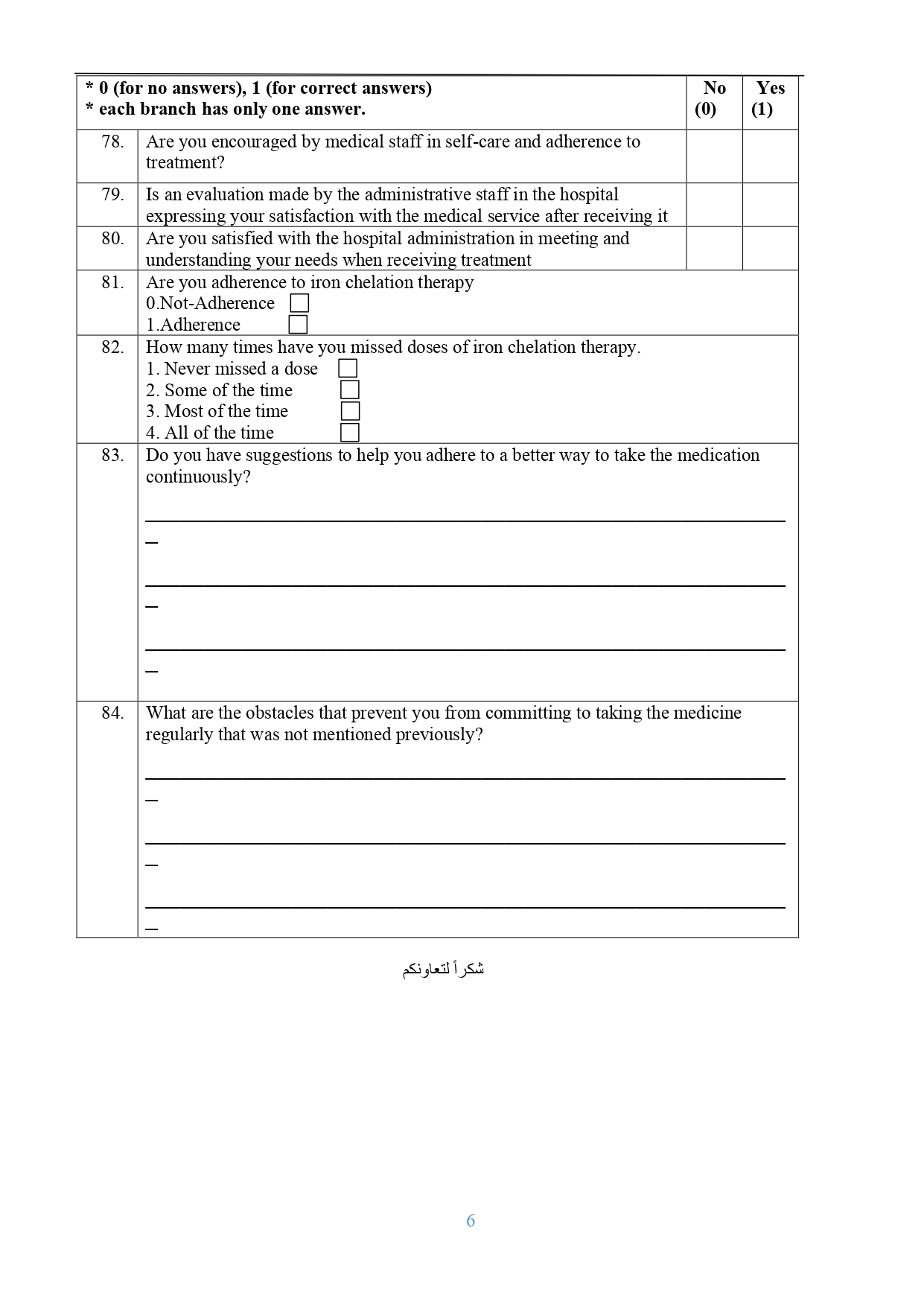


**Supplementary Material S1**: Structured questionnaire used for data collection. It includes items related to sociodemographic characteristics, disease knowledge, and adherence to iron chelation therapy. The questionnaire was developed in Arabic and validated for the local context.
